# Supplementary figures and images for: Succession of the turkey gastrointestinal bacterial microbiome related to weight gain
Source: PeerJ. 2013 Dec 23;1:e237. doi: 10.7717/peerj.237 (PMC3883494; doi:10.7717/peerj.237)

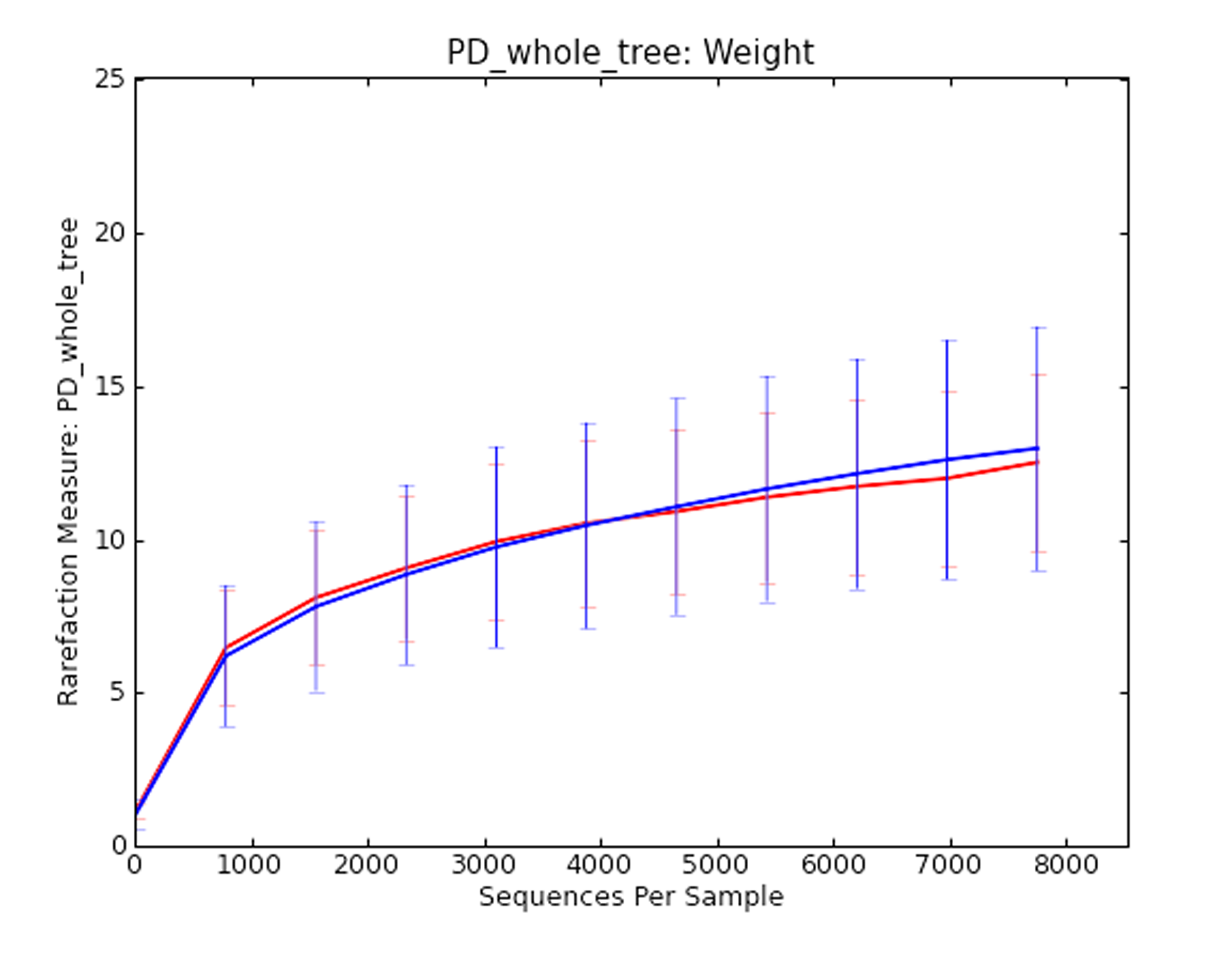

Supplement: Figure S1 — Figure was generated based upon whole-tree phylogenetic diversity. [file peerj-01-237-s001.png]

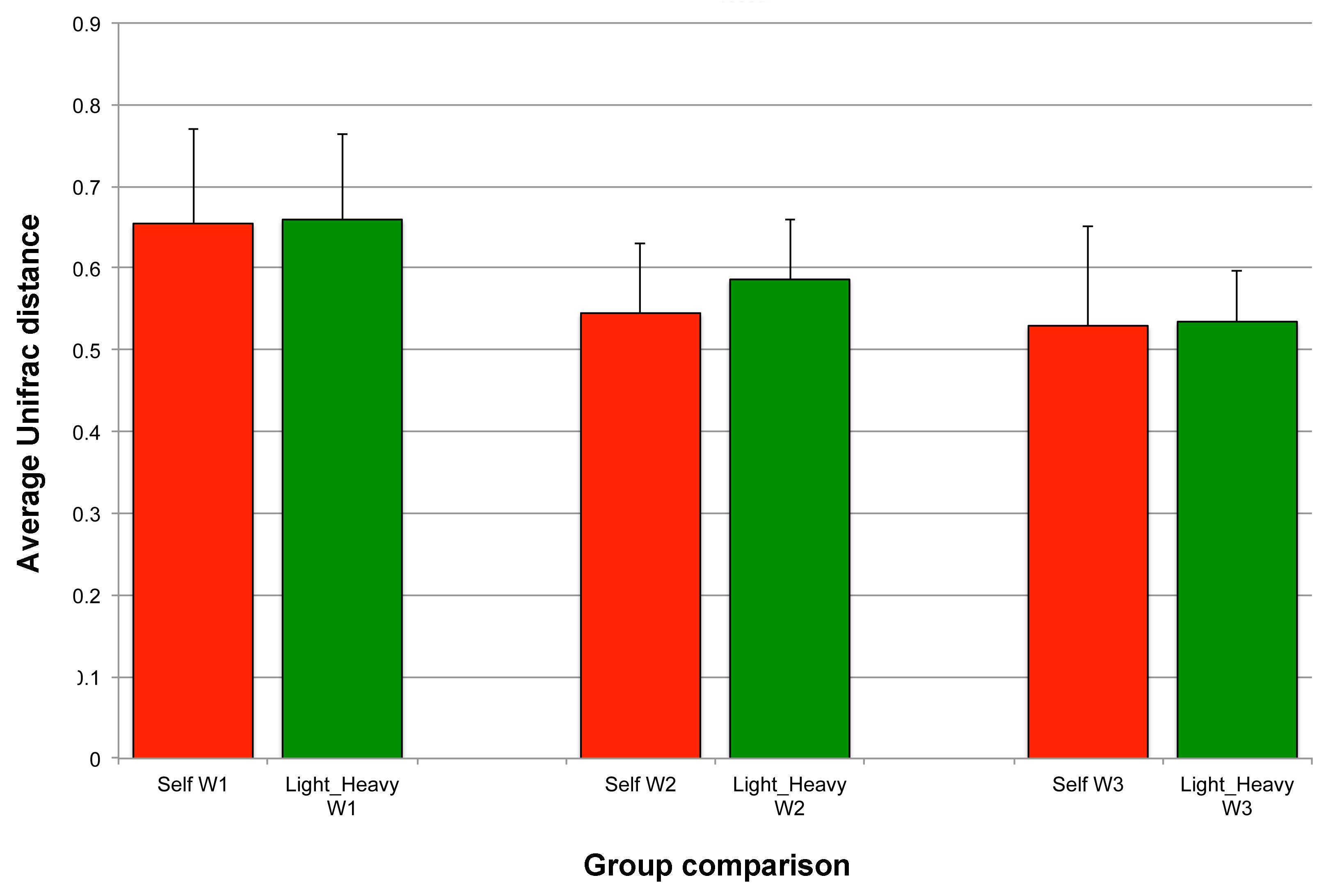

Supplement: Figure S2 — For each timepoint (W), average Unifrac distances for self (red) are compared to Light versus Heavy flocks (green). [file peerj-01-237-s002.png]

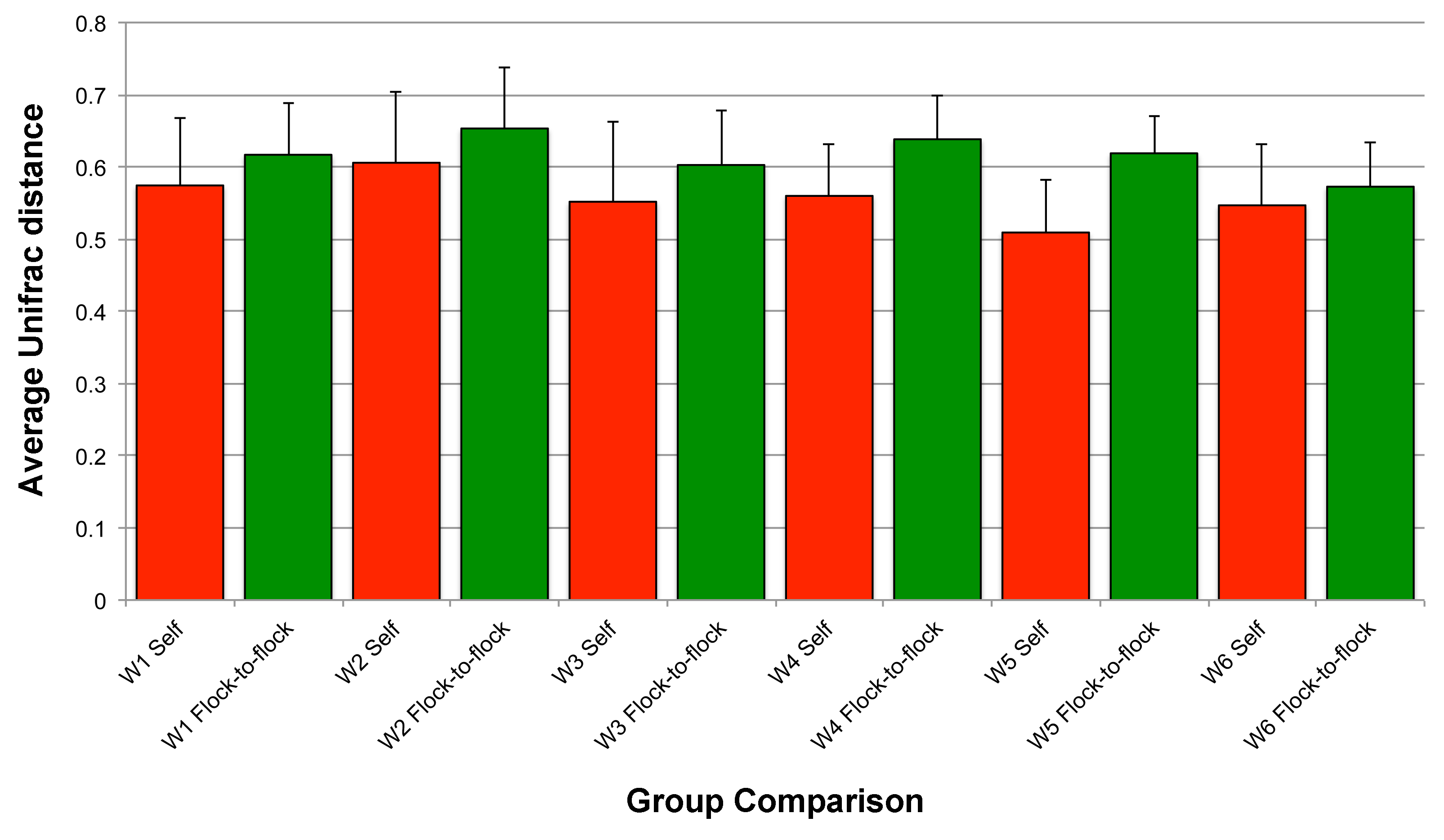

Supplement: Figure S3 — For each timepoint (W), average Unifrac distances are displayed for self (red) and between flocks (green). [file peerj-01-237-s003.png]

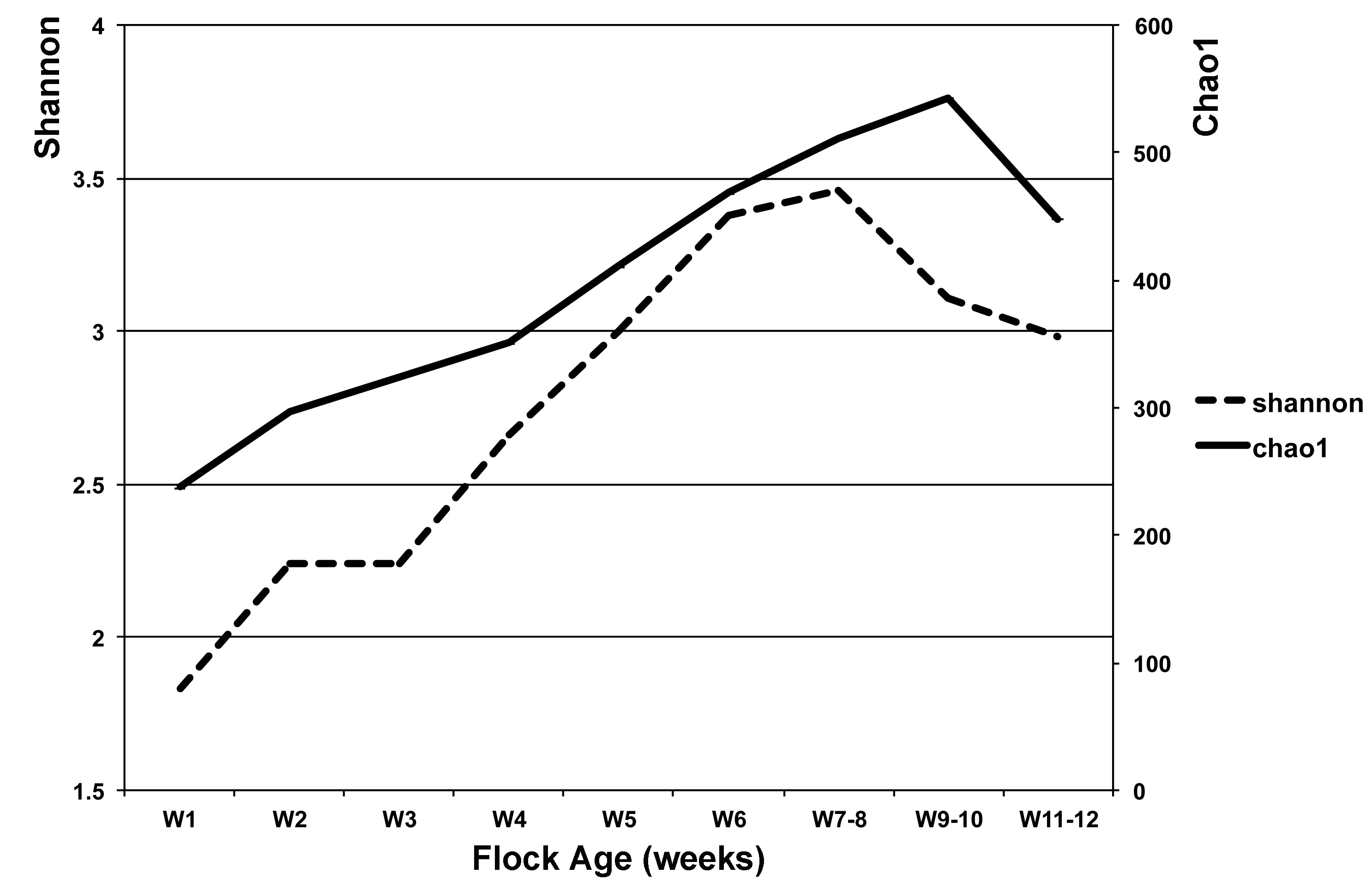

Supplement: Figure S4 — At each timepoint (W), Shannon index is displayed on the left axis (dashed line) and Chao1 estimator is shown on the right axis (solid line). [file peerj-01-237-s004.png]

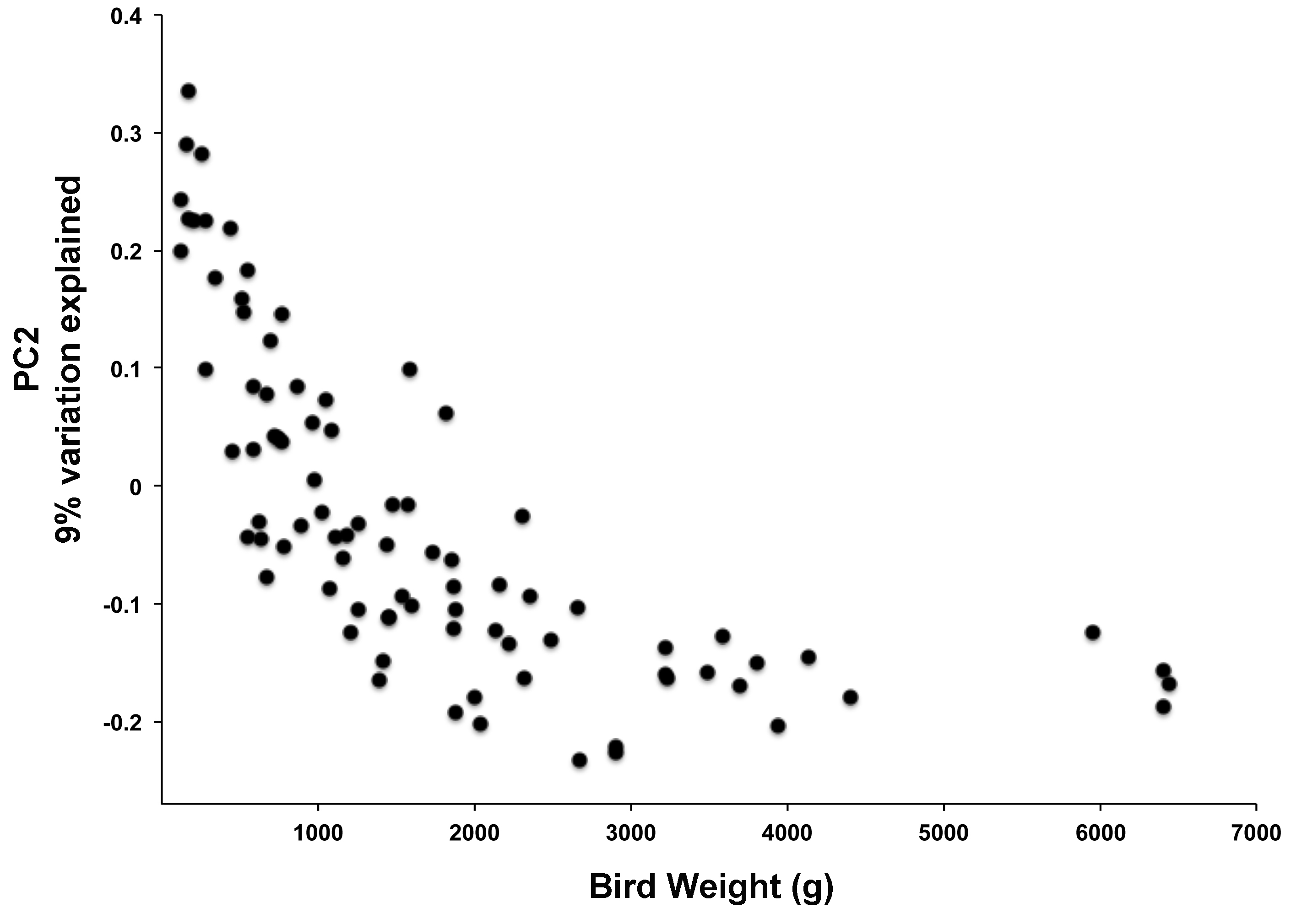

Supplement: Figure S5 — PCoA axis PC2 was plotted against total bird weight in grams. [file peerj-01-237-s005.png]

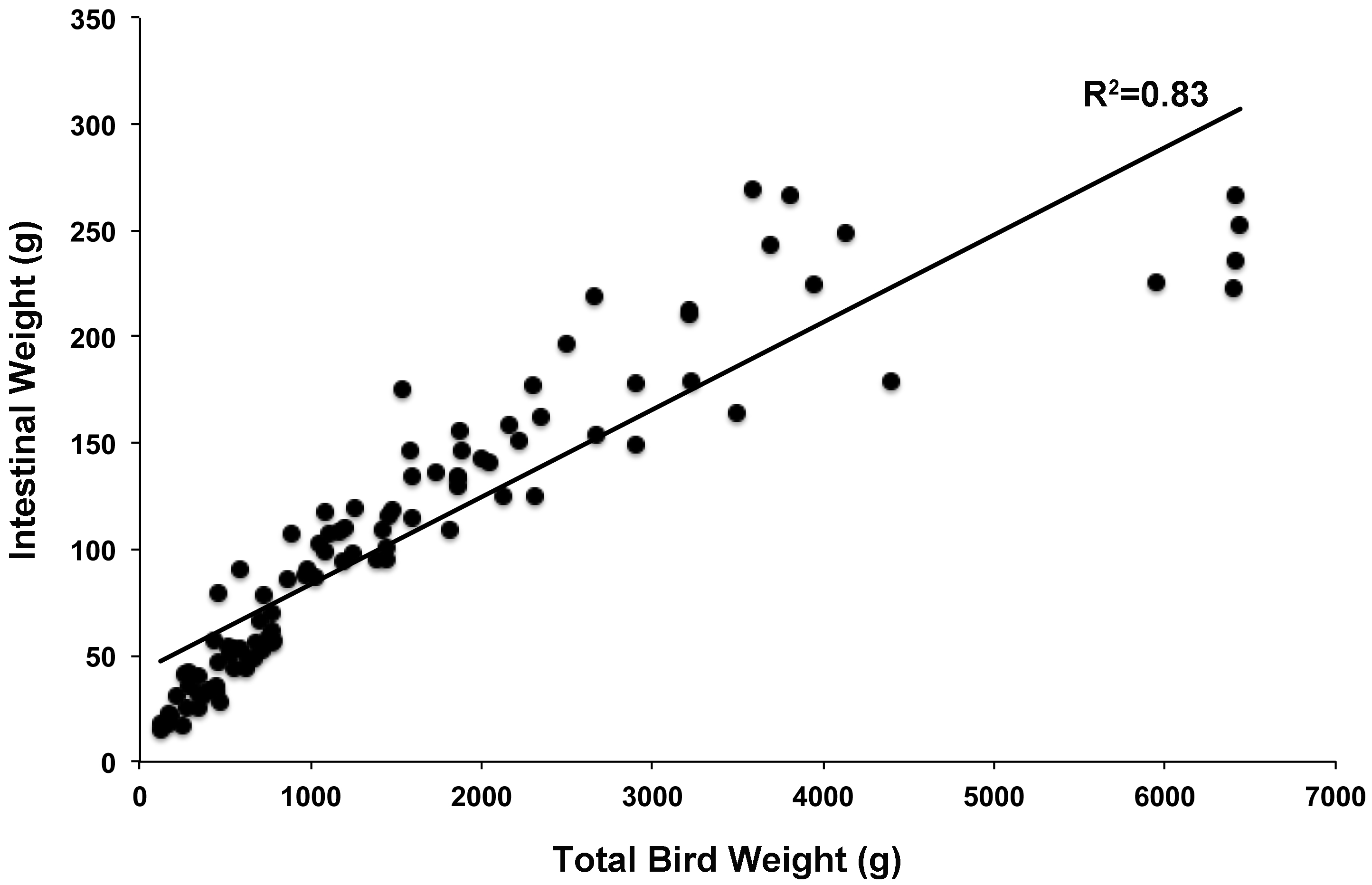

Supplement: Figure S6 [file peerj-01-237-s006.png]
