# Supplementary material for: Succession of the turkey gastrointestinal bacterial microbiome related to weight gain
Source: PeerJ. 2013 Dec 23;1:e237. doi: 10.7717/peerj.237 (PMC3883494; doi:10.7717/peerj.237)
Supplement: Table S1 [file peerj-01-237-s007.docx]

Table S1. Alpha diversity metrics for samples sequenced in this study.

| Sample | chao1 | observed_species | shannon | simpson | simpson_reciprocal | simpson_e | PD_whole_tree |
| --- | --- | --- | --- | --- | --- | --- | --- |
| CFW11 | 196.1 | 133 | 1.4 | 0.3242 | 1.5 | 0.0232 | 12.2 |
| CFW110 | 313.1 | 190 | 2.5 | 0.6094 | 2.6 | 0.0086 | 12.8 |
| CFW111 | 422.9 | 273 | 3.0 | 0.7539 | 4.1 | 0.0049 | 21.3 |
| CFW112 | 309.8 | 161 | 2.8 | 0.7911 | 4.8 | 0.0079 | 12.4 |
| CFW113 | 345.6 | 155 | 2.9 | 0.8019 | 5.0 | 0.0080 | 9.2 |
| CFW114 | 279.0 | 170 | 2.4 | 0.6690 | 3.0 | 0.0088 | 10.9 |
| CFW115 | 299.6 | 132 | 2.8 | 0.7874 | 4.7 | 0.0096 | 7.8 |
| CFW12 | 141.0 | 48 | 0.2 | 0.0498 | 1.1 | 0.4182 | 4.6 |
| CFW13 | 312.5 | 193 | 3.3 | 0.7813 | 4.6 | 0.0066 | 16.0 |
| CFW14 | 268.0 | 108 | 1.5 | 0.4581 | 1.8 | 0.0202 | 9.0 |
| CFW15 | 123.8 | 78 | 0.6 | 0.1319 | 1.2 | 0.0972 | 6.7 |
| CFW16 | 291.8 | 169 | 2.3 | 0.5172 | 2.1 | 0.0114 | 15.8 |
| CFW17 | 217.5 | 95 | 0.7 | 0.1537 | 1.2 | 0.0685 | 9.7 |
| CFW18 | 240.4 | 124 | 2.0 | 0.5814 | 2.4 | 0.0139 | 10.9 |
| CFW19 | 260.7 | 122 | 1.3 | 0.3166 | 1.5 | 0.0259 | 11.9 |
| F1W1 | 88.7 | 58 | 0.2 | 0.0452 | 1.0 | 0.3812 | 7.5 |
| F1W2 | 561.7 | 305 | 3.5 | 0.7886 | 4.7 | 0.0042 | 20.9 |
| F1W3 | 411.2 | 296 | 3.8 | 0.8313 | 5.9 | 0.0041 | 21.3 |
| CFW21 | 296.3 | 93 | 1.8 | 0.6484 | 2.8 | 0.0166 | 6.9 |
| CFW210 | 407.3 | 181 | 2.1 | 0.6294 | 2.7 | 0.0088 | 15.2 |
| CFW22 | 221.5 | 103 | 0.7 | 0.1636 | 1.2 | 0.0593 | 10.0 |
| CFW23 | 287.0 | 127 | 1.5 | 0.3991 | 1.7 | 0.0197 | 11.9 |
| CFW24 | 232.6 | 101 | 1.9 | 0.6802 | 3.1 | 0.0146 | 6.9 |
| CFW25 | 332.9 | 215 | 3.3 | 0.8026 | 5.1 | 0.0058 | 18.6 |
| CFW26 | 189.6 | 104 | 1.8 | 0.6107 | 2.6 | 0.0157 | 6.9 |
| CFW27 | 168.4 | 68 | 1.0 | 0.3474 | 1.5 | 0.0423 | 5.9 |
| CFW28 | 172.1 | 91 | 1.3 | 0.4700 | 1.9 | 0.0234 | 5.4 |
| CFW29 | 243.3 | 125 | 1.4 | 0.5315 | 2.1 | 0.0151 | 9.6 |
| F2W1 | 432.0 | 315 | 4.3 | 0.8979 | 9.8 | 0.0035 | 21.9 |
| F2W2 | 513.6 | 267 | 3.3 | 0.8020 | 5.0 | 0.0047 | 19.7 |
| F2W3 | 475.0 | 295 | 3.4 | 0.7671 | 4.3 | 0.0044 | 21.3 |
| CFW31 | 262.1 | 124 | 1.9 | 0.6114 | 2.6 | 0.0132 | 8.0 |
| CFW310 | 399.4 | 227 | 3.3 | 0.6849 | 3.2 | 0.0064 | 17.5 |
| CFW32 | 536.1 | 282 | 2.6 | 0.5411 | 2.2 | 0.0066 | 21.3 |
| CFW33 | 169.6 | 89 | 0.3 | 0.0451 | 1.0 | 0.2492 | 7.2 |
| CFW34 | 363.8 | 159 | 1.1 | 0.2454 | 1.3 | 0.0256 | 12.8 |
| CFW35 | 231.9 | 119 | 1.6 | 0.5856 | 2.4 | 0.0143 | 10.2 |
| CFW36 | 388.0 | 150 | 1.5 | 0.3685 | 1.6 | 0.0181 | 11.6 |
| CFW37 | 505.8 | 225 | 2.6 | 0.6247 | 2.7 | 0.0071 | 17.8 |
| CFW38 | 264.5 | 141 | 1.0 | 0.2068 | 1.3 | 0.0343 | 12.6 |
| CFW39 | 376.9 | 276 | 4.7 | 0.9061 | 10.7 | 0.0040 | 21.5 |
| CFW41 | 343.1 | 175 | 2.4 | 0.6879 | 3.2 | 0.0083 | 11.1 |
| CFW410 | 248.0 | 181 | 2.6 | 0.5459 | 2.2 | 0.0101 | 17.2 |
| CFW42 | 226.0 | 165 | 1.6 | 0.3448 | 1.5 | 0.0176 | 15.2 |
| CFW43 | 436.8 | 210 | 2.7 | 0.7396 | 3.8 | 0.0064 | 14.2 |
| CFW44 | 353.5 | 151 | 2.5 | 0.7299 | 3.7 | 0.0091 | 11.2 |
| CFW45 | 252.5 | 80 | 2.0 | 0.6210 | 2.6 | 0.0201 | 8.8 |
| CFW46 | 415.1 | 181 | 2.5 | 0.7046 | 3.4 | 0.0078 | 14.1 |
| CFW47 | 335.4 | 169 | 2.7 | 0.7552 | 4.1 | 0.0078 | 15.1 |
| CFW48 | 316.2 | 194 | 2.9 | 0.7711 | 4.4 | 0.0067 | 12.3 |
| CFW49 | 400.7 | 197 | 3.3 | 0.8385 | 6.2 | 0.0061 | 13.6 |
| F4W1 | 327.7 | 209 | 3.5 | 0.8747 | 8.0 | 0.0055 | 15.6 |
| F4W2 | 491.5 | 295 | 3.3 | 0.7498 | 4.0 | 0.0045 | 21.9 |
| F4W3 | 399.6 | 245 | 3.2 | 0.7813 | 4.6 | 0.0052 | 18.2 |
| CFW51 | 418.0 | 196 | 1.8 | 0.4997 | 2.0 | 0.0102 | 14.1 |
| CFW510 | 394.3 | 196 | 2.4 | 0.6275 | 2.7 | 0.0081 | 15.6 |
| CFW52 | 403.2 | 244 | 2.7 | 0.6011 | 2.5 | 0.0068 | 20.7 |
| CFW53 | 331.3 | 201 | 2.4 | 0.6746 | 3.1 | 0.0074 | 15.0 |
| CFW54 | 714.0 | 291 | 3.9 | 0.8636 | 7.3 | 0.0040 | 20.8 |
| CFW55 | 389.1 | 172 | 2.3 | 0.6328 | 2.7 | 0.0092 | 13.4 |
| CFW56 | 281.3 | 142 | 2.2 | 0.6782 | 3.1 | 0.0104 | 11.6 |
| CFW57 | 423.8 | 241 | 3.0 | 0.7814 | 4.6 | 0.0053 | 17.8 |
| CFW58 | 270.7 | 135 | 1.2 | 0.3091 | 1.4 | 0.0240 | 11.5 |
| CFW59 | 422.7 | 208 | 2.2 | 0.5499 | 2.2 | 0.0087 | 16.6 |
| F5W1 | 176.0 | 131 | 2.0 | 0.5988 | 2.5 | 0.0127 | 11.1 |
| F5W2 | 256.2 | 173 | 3.2 | 0.7570 | 4.1 | 0.0076 | 14.8 |
| F5W3 | 235.0 | 180 | 3.2 | 0.8104 | 5.3 | 0.0069 | 15.2 |
| CFW61 | 296.4 | 158 | 2.1 | 0.6562 | 2.9 | 0.0096 | 12.8 |
| CFW62 | 536.0 | 298 | 4.6 | 0.9200 | 12.5 | 0.0036 | 22.1 |
| CFW63 | 512.7 | 305 | 4.7 | 0.9229 | 13.0 | 0.0036 | 23.2 |
| CFW64 | 580.4 | 286 | 3.7 | 0.8423 | 6.3 | 0.0042 | 19.6 |
| CFW65 | 431.1 | 316 | 4.5 | 0.8778 | 8.2 | 0.0036 | 23.1 |
| CFW66 | 356.0 | 170 | 1.7 | 0.4261 | 1.7 | 0.0138 | 12.3 |
| CFW67 | 413.0 | 207 | 3.1 | 0.7924 | 4.8 | 0.0061 | 15.5 |
| CFW68 | 411.0 | 216 | 2.8 | 0.6726 | 3.1 | 0.0069 | 15.7 |
| CFW69 | 637.9 | 375 | 4.7 | 0.9211 | 12.7 | 0.0029 | 22.7 |
| F6W1 | 202.0 | 117 | 1.4 | 0.3602 | 1.6 | 0.0237 | 11.0 |
| F6W2 | 256.1 | 189 | 2.7 | 0.7053 | 3.4 | 0.0075 | 16.6 |
| F6W3 | 328.6 | 204 | 3.3 | 0.8244 | 5.7 | 0.0059 | 18.1 |
| CFW71 | 347.6 | 155 | 2.2 | 0.6415 | 2.8 | 0.0101 | 10.3 |
| CFW72 | 536.8 | 315 | 3.4 | 0.7472 | 4.0 | 0.0042 | 18.6 |
| CFW73 | 592.6 | 349 | 4.7 | 0.9239 | 13.1 | 0.0031 | 21.8 |
| CFW74 | 412.4 | 214 | 2.5 | 0.6417 | 2.8 | 0.0073 | 15.3 |
| CFW75 | 451.6 | 246 | 3.5 | 0.8579 | 7.0 | 0.0047 | 17.0 |
| F7W1 | 296.1 | 142 | 1.1 | 0.2501 | 1.3 | 0.0282 | 13.3 |
| F7W2 | 234.0 | 135 | 2.9 | 0.8288 | 5.8 | 0.0089 | 11.5 |
| F7W3 | 195.6 | 88 | 2.5 | 0.7810 | 4.6 | 0.0146 | 9.5 |
| F8W1 | 250.9 | 136 | 2.9 | 0.8043 | 5.1 | 0.0091 | 12.6 |
| F8W2 | 383.6 | 256 | 4.3 | 0.8739 | 7.9 | 0.0045 | 20.7 |
| F8W3 | 331.5 | 159 | 3.0 | 0.7206 | 3.6 | 0.0087 | 14.4 |
| CFW91 | 390.6 | 179 | 3.0 | 0.8316 | 5.9 | 0.0067 | 13.6 |
| CFW92 | 471.3 | 254 | 3.2 | 0.8211 | 5.6 | 0.0048 | 17.9 |
| CFW93 | 380.0 | 218 | 2.6 | 0.6232 | 2.7 | 0.0074 | 16.9 |
| CFW94 | 372.8 | 228 | 2.9 | 0.7439 | 3.9 | 0.0059 | 16.4 |
| CFW95 | 449.7 | 251 | 2.8 | 0.7266 | 3.7 | 0.0055 | 17.9 |
| RFW101 | 714.5 | 336 | 3.3 | 0.7810 | 4.6 | 0.0038 | 19.7 |
| RFW102 | 645.4 | 359 | 3.4 | 0.7757 | 4.5 | 0.0036 | 23.7 |
| RFW103 | 649.2 | 366 | 3.5 | 0.7932 | 4.8 | 0.0034 | 20.5 |
| RFW104 | 750.2 | 326 | 3.3 | 0.7983 | 5.0 | 0.0038 | 20.7 |
| RFW105 | 607.2 | 282 | 3.1 | 0.8061 | 5.2 | 0.0044 | 17.7 |
| RFW11 | 226.2 | 99 | 2.0 | 0.6575 | 2.9 | 0.0154 | 9.6 |
| RFW12 | 305.4 | 149 | 2.5 | 0.7382 | 3.8 | 0.0091 | 11.5 |
| RFW121 | 441.2 | 255 | 2.9 | 0.7330 | 3.7 | 0.0054 | 16.7 |
| RFW122 | 700.6 | 365 | 3.7 | 0.8094 | 5.2 | 0.0034 | 24.4 |
| RFW123 | 368.2 | 191 | 1.8 | 0.4222 | 1.7 | 0.0124 | 14.2 |
| RFW124 | 636.7 | 363 | 4.4 | 0.9047 | 10.5 | 0.0030 | 24.3 |
| RFW125 | 680.8 | 283 | 3.1 | 0.7770 | 4.5 | 0.0045 | 15.8 |
| RFW13 | 151.2 | 75 | 0.4 | 0.0998 | 1.1 | 0.1336 | 8.6 |
| RFW14 | 204.6 | 128 | 2.3 | 0.7003 | 3.3 | 0.0112 | 11.9 |
| RFW15 | 201.0 | 99 | 1.6 | 0.5857 | 2.4 | 0.0172 | 9.0 |
| RFW21 | 398.3 | 281 | 3.7 | 0.8197 | 5.5 | 0.0043 | 18.3 |
| RFW22 | 190.5 | 111 | 1.8 | 0.5885 | 2.4 | 0.0153 | 10.5 |
| RFW23 | 200.9 | 85 | 0.9 | 0.2647 | 1.4 | 0.0444 | 7.6 |
| RFW24 | 222.9 | 116 | 1.8 | 0.5744 | 2.3 | 0.0150 | 10.6 |
| RFW25 | 275.8 | 145 | 1.2 | 0.3050 | 1.4 | 0.0226 | 12.1 |
| RFW31 | 308.3 | 163 | 1.4 | 0.3870 | 1.6 | 0.0159 | 14.6 |
| RFW32 | 268.1 | 138 | 0.6 | 0.1161 | 1.1 | 0.0624 | 13.6 |
| RFW33 | 222.7 | 135 | 0.7 | 0.1233 | 1.1 | 0.0601 | 13.3 |
| RFW34 | 249.8 | 164 | 2.6 | 0.7416 | 3.9 | 0.0082 | 13.4 |
| RFW35 | 294.8 | 193 | 2.6 | 0.5997 | 2.5 | 0.0086 | 14.5 |
| RFW41 | 323.2 | 207 | 2.9 | 0.7445 | 3.9 | 0.0065 | 18.2 |
| RFW42 | 475.3 | 344 | 4.9 | 0.9205 | 12.6 | 0.0032 | 23.8 |
| RFW43 | 389.1 | 166 | 1.5 | 0.3891 | 1.6 | 0.0155 | 14.8 |
| RFW44 | 410.1 | 208 | 3.2 | 0.8278 | 5.8 | 0.0058 | 14.3 |
| RFW45 | 340.0 | 160 | 2.4 | 0.6897 | 3.2 | 0.0091 | 12.6 |
| RFW51 | 450.0 | 261 | 3.7 | 0.8154 | 5.4 | 0.0047 | 20.1 |
| RFW52 | 485.2 | 338 | 4.9 | 0.9332 | 15.0 | 0.0032 | 21.0 |
| RFW53 | 370.4 | 261 | 3.7 | 0.7859 | 4.7 | 0.0049 | 19.1 |
| RFW54 | 398.8 | 257 | 4.2 | 0.9036 | 10.4 | 0.0043 | 18.1 |
| RFW55 | 402.4 | 249 | 2.6 | 0.6990 | 3.3 | 0.0057 | 17.1 |
| RFW61 | 395.1 | 175 | 2.4 | 0.7035 | 3.4 | 0.0081 | 14.9 |
| RFW62 | 477.6 | 338 | 4.5 | 0.9179 | 12.2 | 0.0032 | 21.8 |
| RFW63 | 511.3 | 293 | 3.1 | 0.7103 | 3.5 | 0.0048 | 19.5 |
| RFW64 | 480.0 | 255 | 3.0 | 0.7503 | 4.0 | 0.0052 | 15.9 |
| RFW65 | 521.2 | 262 | 2.3 | 0.5510 | 2.2 | 0.0069 | 16.6 |
| RFW81 | 569.4 | 311 | 3.8 | 0.8678 | 7.6 | 0.0037 | 19.8 |
| RFW82 | 715.1 | 372 | 3.9 | 0.8516 | 6.7 | 0.0032 | 23.7 |
| RFW83 | 453.3 | 314 | 4.3 | 0.9070 | 10.8 | 0.0035 | 21.4 |
| RFW84 | 615.9 | 321 | 3.4 | 0.7485 | 4.0 | 0.0042 | 21.5 |
| RFW85 | 422.8 | 224 | 2.9 | 0.7459 | 3.9 | 0.0060 | 16.1 |
